# Possible Roles of Proinflammatory Signaling in Keratinocytes Through Aryl Hydrocarbon Receptor Ligands for the Development of Squamous Cell Carcinoma

**DOI:** 10.3389/fimmu.2020.534323

**Published:** 2020-10-16

**Authors:** Yota Sato, Taku Fujimura, Takanori Hidaka, Chunbing Lyu, Kayo Tanita, Shigeto Matsushita, Masayuki Yamamoto, Setsuya Aiba

**Affiliations:** ^1^Department of Dermatology, Tohoku University Graduate School of Medicine, Sendai, Japan; ^2^Department of Dermato-Oncology/Dermatology, National Hospital Organization Kagoshima Medical Center, Kagoshima, Japan; ^3^Department of Medical Biochemistry, Tohoku University Graduate School of Medicine, Sendai, Japan

**Keywords:** aryl hydrocarbon receptor, cutaneous SCC, IL-17, carcinogenesis, proinflammatory cytokines

## Abstract

Aryl hydrocarbon receptor (AhR) provides a deeper insight into the pathogenesis of cutaneous squamous cell carcinoma (cSCC). AhR ligands, such as 6-formylindolo[3,2-b] carbazole (FICZ), and 7,12-Dimethylbenz[a]anthracene (DMBA), constitute major substrates for the cytochrome P450 (CYP) family, and influence the expression of various cytokine genes, including *IL-17* and *IL-23*-related genes via the AhR. On the other hand, proinflammatory cytokines could drive tumor progression through the TRAF-ERK5 signaling pathway in cSCC. From the above findings, we hypothesized that AhR ligands might enhance the mRNA expression of proinflammatory cytokines via the AhR, leading to the development of cSCC. The purpose of this study was to investigate (1) the immunomodulatory effects of FICZ and DMBA on normal human keratinocytes (NHKCs), focusing on IL-17, and related cytokines/chemokines (IL-23, IL-36γ, and CCL20), (2) the expression of these factors in AhR-dependent pathways using a two-stage chemically induced skin carcinogenesis mouse model, and (3) the expression of these factors in lesion-affected skin in cSCC. Both FICZ and DMBA augmented the expression of CYP1A1, p19, CCL20, and IL-36γ mRNA in NHKCs *in vitro*. Moreover, the mRNA expression of these proinflammatory factors, as well as IL-17, in mouse cSCC is significantly decreased in the AhR-(fl/fl) Krt5-(Cre) mice compared to wild type mice, leading to a decrease in the number of developed cSCC lesions. Furthermore, CCL20, IL-23, as well as IL-17, are detected in the lesion-affected skin of cSCC patients. Our study demonstrates a possible mechanism for the development of cSCC involving AhR-mediated signaling by epidermal keratinocytes and recruitment of Th17 cells.

## Introduction

Cutaneous squamous cell carcinoma (cSCC) is the second most common type of non-melanoma skin cancer, and its risk factors have been widely reported (1, 2). For example, the precursor of cSCC is intraepithelial UV-induced damage, which can develop into actinic keratosis (AK) (3). Indeed, AK and SCC possess a similar genetic profile, such as alterations in the p53 gene (3), suggesting that AK could sequentially develop into cSCC. On the other hands, chemical exposure is another risk of cSCC. Recently, Chahal et al. reported a two-stage genome-wide association study for cSCC (4), suggesting the genome-wide significance of seven pigmentation-related loci and four susceptibility loci, including aryl hydrocarbon receptor (AhR) and IRF4. AhR is a dioxin receptor involved in anti-apoptotic pathways and progression of melanoma, whereas *IRF4* encodes a key transcription factor that controls M*2* macrophage polarization (5). The authors concluded that these susceptibility loci provide a deeper insight into the pathogenesis of cSCC (4).

The exposure of keratinocytes to ultraviolet (UV) radiation leads to the intracellular production of 6-formylindolo[3,2-b]carbazole (FICZ), which exhibits high affinity for AhR (6, 7). FICZ is a tryptophan oxidation product formed by exposure to UV, and is metabolized by CYP1A1 (7). FICZ is a major substrate for the cytochrome P450 (CYP) family, and affects the expression of various genes via the AhR (8–10). Despite various reports about the physical and chemical properties of FICZ, its association with cSCC remains unclear.

7,12-Dimethylbenz[a]anthracene (DMBA), another type of AhR ligand that is typically found in cigarette smoke, is widely known to induce cutaneous SCC in mouse skin together with 12-O-Tetradecanoylphorbol 13-acetate (TPA) (11, 12). The majority of DMBA-induced SCCs possess mutations in oncogenes including Hras, Kras, and Rras (13, 14), which are detected in human SCC located in cervical, esophageal, and lung tissues, among others. Although these previous reports suggested the significance of oncogenic mutations caused by AhR ligands, the immunomodulatory effects of the AhR in cSCC are still unknown. Notably, AhRs are highly activated in Th17 cells (14), and AhR ligands enhance the differentiation of Th17 cells and IL-22 production via the AhR (15). Considering that IL-17 could drive the tumor progression through TRAF-ERK5 pathways in cSCC (16), we hypothesized that DMBA could induce IL-17 production from keratinocytes to drive the proliferation of keratinocytes, leading to the development of cSCC. In this report, we investigated the significance of AhR signaling in keratinocytes for the development of cSCC using a two-stage chemically induced skin carcinogenesis mouse model and human cSCC samples.

## Materials and Methods

### Ethics Statement for Animal and Human Experiments

The protocol for the animal study was approved by the ethics committee at Tohoku University Graduate School of Medicine for Animal Experimentation, Sendai, Japan (permit number: 2017MdLMO-342-2). The research complied with the Tohoku University Graduate School of Medicine’s Animal Experimentation Ethics guidelines and policies. All surgeries were performed under sodium pentobarbital anesthesia, and all efforts were made to minimize suffering. The protocol for the human study was approved by the ethics committee at Tohoku University Graduate School of Medicine, Sendai, Japan (permit number: 2017-1-430), and Kagoshima Medical Center, Japan (permit number 29-2, 30-08).

### Animals and Melanoma Cell Line

C57BL/6 mice and BALB/c mice (5 to 8 weeks old) were purchased from Japan Shizuoka Laboratory Animal Center (Shizuoka, Japan) and housed in the animal facility at the Tohoku University Graduate School of Medicine. *Ahr*^fl/fl^ mice and *Krt5*-Cre mice were kindly provided by Department of Medical Biochemistry, Tohoku University Graduate School of Medicine, Sendai, Japan (6).

### Reagents

The following antibodies (Abs) were used for immunohistochemical staining: mouse monoclonal Abs (LifeSpan BioScience, Seattle, WA, United States) against human CCL20 and human IL-36γ, rabbit polyclonal Abs (LifeSpan BioScience) against human CYP1A1 and human IL-23, and a goat polyclonal Ab (R&D Systems, Minneapolis, MN, United States) against human IL-17. The following antibodies were used for immunofluorescence (IF): mouse anti-human CD163 phycoerythrin-conjugated monoclonal antibody (R&D Systems), rabbit polyclonal anti-CCL22 antibody (R&D Systems), rabbit polyclonal anti-CXCL5 antibody (Lifespan Bioscience, Seattle, WA, United States), mouse anti-CXCL10 antibody (Lifespan Bioscience), Alexa Fluor 488-conjugated anti-mouse rat immunoglobulin (Ig)G (Abcam, Tokyo, Japan), and Alexa Fluor 488-conjugated anti-rabbit goat IgG (Abcam).

### Tissue Samples and Immunohistochemical Staining

We collected archived formalin-fixed paraffin-embedded skin specimens and cryosections from cutaneous SCC patients treated in the Department of Dermatology at Tohoku University Graduate School of Medicine, Sendai, Japan, and Department of Dermato-Oncology/Dermatology at Kagoshima Medical Center, Kagoshima Japan. We employed immunohistochemical staining for 10 cases of squamous cell carcinoma and 10 cases of AK (Table 1). For cryosections, each sample was frozen in optimal cutting temperature embedding medium, and 6-μm sections were fixed with cold acetone for 30 min and then blocked with IF buffer (PBS, 5% bovine serum albumin). Thereafter, each section was incubated with the relevant antibodies. The slides were mounted in DAPI Fluoromount-G (Southern Biotech, Birmingham, AL, United States) and examined using a Zeiss LSM 700 microscope equipped with a SPOT digital camera (Zeiss Japan, Tokyo, Japan).

**TABLE 1:** Characteristics of patients with cSCC and actinic keratosis.

	Age	Stage	Location	Histology
**Invasive**				
Case 1	31–40	T2N0M0 stage II	Scalp	Well differentiated
Case 2	51–60	T1N0M0 stage I	Cheek	Well differentiated
Case 3	71–80	T2N0M0 stage II	Scalp	Well differentiated
Case 4	61–70	T1N0M0 stage I	Forearm	Well differentiated
Case 5	71–80	T1N0M0 stage I	Nose	Well differentiated
Case 6	71–80	T1N0M0 stage I	Cheek	Well differentiated
Case 7	91–100	T2N0M0 stage II	Scalp	Well differentiated
Case 8	61–70	T2N0M0 stage II	Lower leg	Well differentiated
Case 9	61–70	T3N0M0 stage III	Scalp	Well differentiated
Case 10	71–80	T1N0M0 stage I	Scalp	Well differentiated
Case 11	61–70	T1N0M0 stage I	Penis	Well differentiated
Case 12	31–4-	T1N0M0 stage I	Scalp	Well differentiated
***In situ***				
Case 1	71–80	TisN0M0 stage 0	Forearm	Actinic keratosis
Case 2	81–90	TisN0M0 stage 0	Forearm	Actinic keratosis
Case 3	71–80	TisN0M0 stage 0	Ear	Actinic keratosis
Case 4	81–90	TisN0M0 stage 0	Cheek	Actinic keratosis
Case 5	61–70	TisN0M0 stage 0	Cheek	Actinic keratosis
Case 6	91–100	TisN0M0 stage 0	Medial canthu	Actinic keratosis
Case 7	81–90	TisN0M0 stage 0	Scalp	Actinic keratosis
Case 8	81–90	TisN0M0 stage 0	Preauricle	Actinic keratosis
Case 9	81–90	TisN0M0 stage 0	Cheek	Actinic keratosis
Case 10	91–100	TisN0M0 stage 0	Forearm	Actinic keratosis
Case 11	71–80	TisN0M0 stage 0	Scalp	Actinic keratosis
Case 12	91–100	TisN0M0 stage 0	Cheek	Actinic keratosis

### Cell Culture and Stimulation

Normal human epidermal keratinocytes (NHKCs; Kurabo, Osaka, Japan) were cultured in HuMedia-KG supplemented with insulin (10 μg/mL), hEGF (0.1 ng/mL), hydrocortisone (0.5 μg/mL), gentamicin (50 μg/mL), amphotericin B (50 μg/mL), and fetal bovine serum (0.4% v/v; Kurabo). Cells were cultured at 37°C in a 5% CO_2_ atmosphere. Upon reaching 80% confluence, cells were treated with FICZ (10 nM), or DMBA (1 μM) for 4 h.

### RNA Extraction, Assessment of RNA Quality, and Reverse Transcription and Quantitative Real-Time PCR

Total RNA was extracted using a RNeasy Micro kit (Qiagen, Courtaboeuf, France) in accordance with the manufacturer’s instructions. The RNA was eluted using 4 μL of RNase-free water. Contaminating genomic DNA was removed by treating extracted RNA with DNase I (RNase-Free DNase Set; Qiagen). Reverse transcription was performed using a SuperScript VILO cDNA Synthesis kit (Invitrogen). Amplification reactions were performed using a Mx 3000P Real-Time Quantitative PCR System (Stratagene, Tokyo, Japan). The thermal cycling conditions were as follows: 3 min for polymerase activation at 95°C, followed by 40 cycles at 95°C for 5 s and 60°C for 20 s. PCR products were maintained at 4°C. Relative mRNA expression levels were calculated for each gene and each time point after normalization against the expression of glyceraldehyde 3-phosphate dehydrogenase (*GAPDH*) mRNA using the ΔΔCt method. Averaged data from at least three independent experiments are shown.

### Cytokine Enzyme-Linked Immunosorbent Assays

Secretion of CCL20 (R&D Systems), IL-36γ (R&D Systems), and IL-23 (R&D Systems) in NHKCs was determined using enzyme-linked immunosorbent assay (ELISA) kits, according to the manufacturer’s instructions.

### Western Blotting

Normal human epidermal keratinocytes were seeded into 12-well plates and cultured as described above. Cells were collected and disrupted in lysis buffer (Cell Signaling Technology, Boston, MA, United States). After adding SDS sample buffer (Cell Signaling Technology), lysates were electrophoretically separated on a 12% polyacrylamide gel (ATTO Corp., Tokyo, Japan). Proteins were electrophoretically transferred onto a polyvinylidene difluoride membrane (Bio-Rad, Hercules, CA, United States). The membrane was blocked in 5% non-fat dry milk in Tris–buffered saline (TBS) with 0.1% Tween-20 (TBST) for 1 h at room temperature. After several washes with TBST, the membrane was incubated overnight at 4°C with primary mouse anti-human IL-36 beta antibody (R and D system; 1:1000), anti-human IL-36 gamma antibody (R and D system; 1:1000), anti-human p19 antibody (Proteintech, Tokyo; 1:1000), or anti-human tublin antibody (Proteintech; 1:1000). The membrane was washed several times in TBST followed by a 1-h incubation with horseradish peroxidase–conjugated goat anti-mouse IgG secondary antibody (Santa Cruz, Dallas, TX, United States).

### Two-Stage Chemically Induced Skin Carcinogenesis Mouse Model

7,12-Dimethylbenz[a]anthracene was purchased from Sigma-Aldrich, Merck Milipore, Billerica, MA, United States, and TPA was purchased from Calbiochem, Merck Milipore, Billerica, MA, United States. DMBA is used as a carcinogen and TPA as a promoter. At 6–8 weeks of age, the backs of the mice were shaved, and 2 days after shaving, DMBA (25 μg per mouse in 200 μL acetone) was applied to shaved dorsal back skin. 3 days after the first DMBA treatment, TPA (10 μg per mouse in 200 μL acetone) was applied. After four rounds of this single DMBA and TPA treatment, the mice were treated with TPA twice weekly for 20 weeks.

For qRT-PCR, the whole tumor was frozen with liquid nitrogen, then crushed with a Cryo-Press (MICROTEC, Chiba, Japan), as described previously (17). Total RNA was extracted using ISOGEN (NIPPON GENE, Tokyo, Japan) according to the manufacturer’s instructions.

### Assessment of Immunohistochemical Staining

The intensity of immunohistochemical staining for each antibody was scored on a semiquantitative scale (Table 1).

### Statistical Analysis

Statistical analysis was performed using the Mann–Whitney *U*-test for comparison of values. The level of significance was set at *p* < 0.05.

## Results

### Expression of CYP1A1 and Aryl Hydrocarbon Receptor and in Cutaneous Cell Carcinoma, Actinic Keratosis and Normal Skin

**FIGURE 1:**
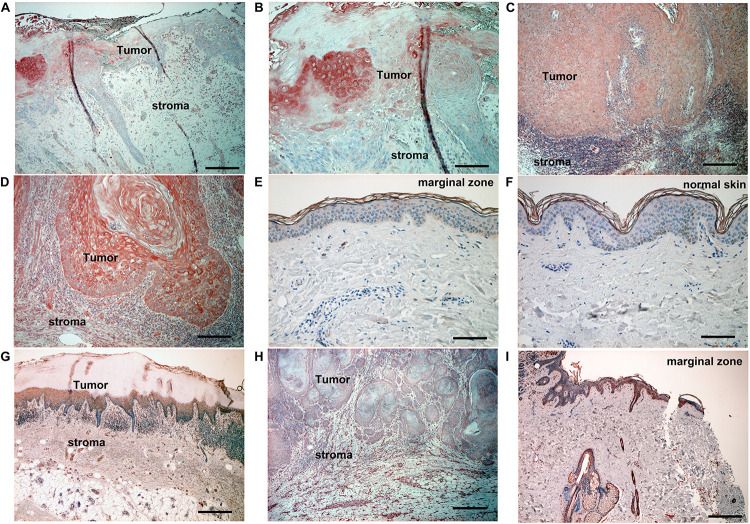
Immunohistochemical analysis of CYP1A1 expression and AhR expression in lesion-affected skin of AK and cSCC. Sections of skin from lesion-affected areas of AK **(A,B,G)**, cSCC **(C,D,H)**, marginal areas around cSCC lesions **(E,I)**, or normal skin ([**F**]; nevus pigmentosus located at back) were deparaffinized and stained using anti-CYP1A1 antibodies **(A–F)**, or AhR antibodies **(G–I)**. The sections were developed with liquid permanent red. Scale bars, 100 μm **(B,D–F)**, 200 μm **(A,C,G–I)**. Representative specimens from analyses of 5 cases of actinic keratosis, 12 cases of cSCC, and 10 cases of nevus pigmentosus are shown.

Since the AhR ligands, FICZ, and DMBA, are reported to promote carcinogenesis in SCC (11), we firstly employed immunohistochemical staining of CYP1A1 and AhR in 10 cases in each condition (cutaneous SCC, AK, and normal skin). Atypical keratinocytes in AK (Figures 1A,B) and cutaneous SCC (Figures 1C,D) expressed CYP1A1, whereas normal keratinocytes between the follicular bulbs in AK (Figure 1A), surgical margin of cutaneous SCC (Figure 1E), or normal skin (Figure 1F) did not express CYP1A1. Atypical keratinocytes in AK (Figure 1G), SCC (Figure 1H), and normal keratinocytes in basal layer (Figure 1I) of epidermis expressed AhR.

### AhR Ligand Increases the Expression of CYP1A1, CCL20, p19, and IL-36γ mRNA in NHKCs

**FIGURE 2:**
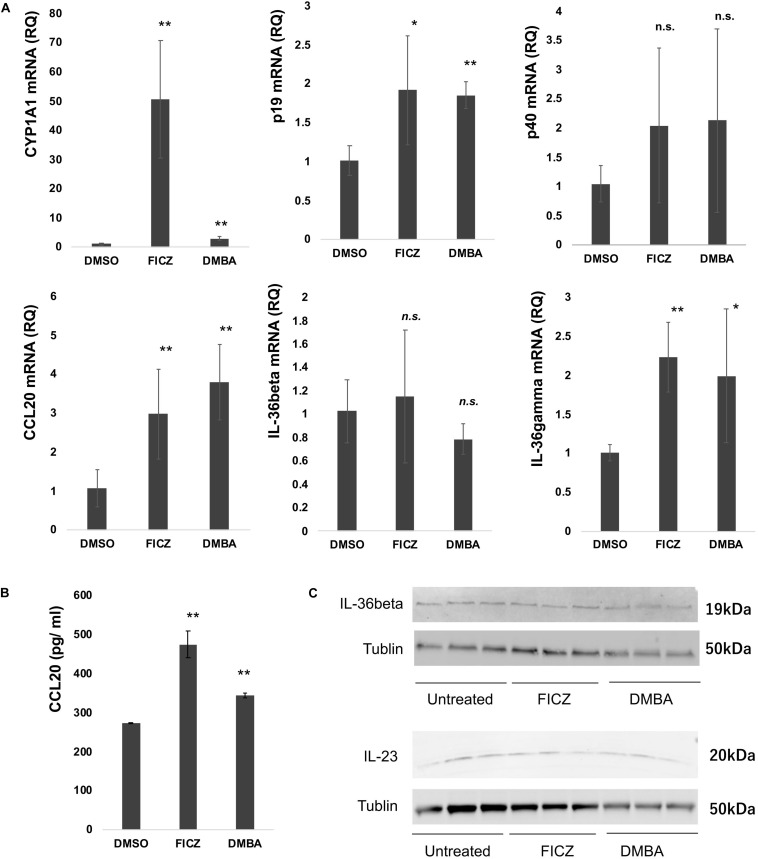
Expression of CYP1A1, cytokine, and chemokine mRNA in normal human keratinocytes (NHKCs) stimulated with FICZ, or DMBA. NHKCs were cultured and treated with FICZ (10 nM), or DMBA (1 μM) as described in the Materials and Methods. At 4 h after stimulation, total RNA was recovered from NHKCs and amplified, labeled, and analyzed. Quantitative real-time PCR was conducted to determine the number of cDNA copies for each factor, and the mRNA expression level relative to that of untreated cells was calculated for each gene and time point after normalization against glyceraldehyde 3-phosphate dehydrogenase using the ΔΔCt method **(A)**. NHKC culture supernatant was harvested as described in the Materials and Methods and analyzed by ELISA **(B)**. Data from each donor were obtained from triplicate assays, and the mean ± SD was calculated. The means of at least three independent experiments are shown. IL-23 and IL-36β production was analyzed by western blotting as described in the Materials and Methods **(C)**. *p < 0.05, **p < 0.01.

Considering that the atypical keratinocytes in SCC and AK express CYP1A1, AhR ligands stimulate NHKCs to increase the production of CYP1A1 as well as proinflammatory cytokines (18), and since IL-17 could drive the tumor progression through TRAF-ERK5 pathways in cSCC (16), we next examined the immunomodulatory effects of FICZ and DMBA on NHKCs, focusing on the expression of CYP1A1, CCL20, p19, p40, IL-36α, IL-36β, and IL-36γ mRNA at 4 h after stimulation *in vitro*. Both FICZ and DMBA augmented the expression of CYP1A1 as well as CCL20, p19, and IL-36γ mRNA (Figure 2). There was no significant increase in p40 and IL-36β mRNA expression (Figure 2).

### FICZ and DMBA Increased the Production of CCL20, IL-36γ and p19 in NHKCs

As the results shown in Figure 2A suggest, FICZ and DMBA significantly increased the expression of CCL20, IL-36γ, and p19 in NHKCs. Therefore, we confirmed the production of CCL20, IL-36γ, and p19 protein in NHKCs treated with FICZ and DMBA *in vitro*. Production of CCL20 was significantly increased by treatment with FICZ and DMBA (Figure 2B). The production of IL-23 and IL-36β was detected by Western blot in each group (Figure 2C). The production of IL-36γ was not detected by ELISA and Western blot in each group.

### AhR Dependency of Two-Stage Chemically Induced Skin Carcinogenesis Mouse Model

**FIGURE 3:**
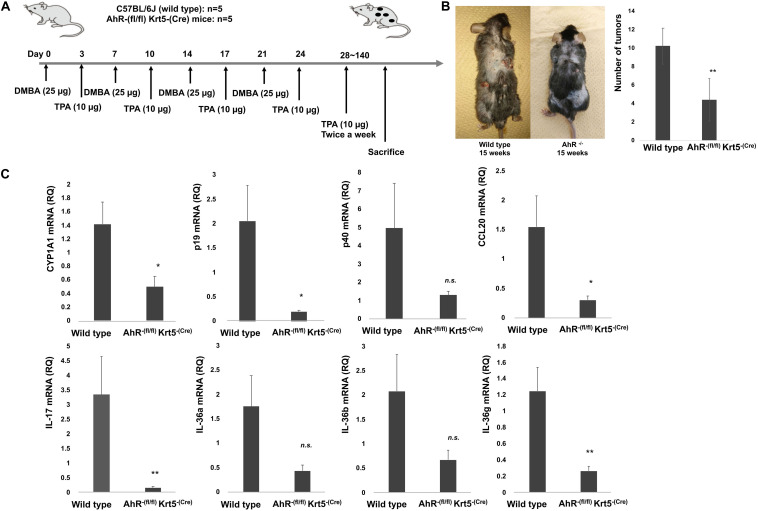
AhR dependency in two-stage chemically induced skin carcinogenesis mouse model. Mouse cSCC in each mouse was induced as described in the Materials and Methods. The schematic representation of model of carcinogenesis **(A)**. The representative figure of DMBA-induced tumor in 5 wild type (AhR+/Krt5+) mouse and 5 AhR-(fl/fl) Krt5-(Cre) mouse **(B)**. The number of developed tumors were counted by two independent researchers, and the average number of induced tumors is calculated **(B)**. Expression of chemokines and cytokines in DMBA-induced tumor was analyzed by quantitative RT-PCR using the ΔΔCt method (*n* = 5). The data from each donor were obtained by triplicate assays, and then the mean ± SD was calculated **(C)**. *Marks a significant (*p* < 0.05) difference. **Marks a significant (*p* < 0.01) difference.

To further examine the immunomodulatory mechanisms responsible for the AhR signal in the carcinogenesis of cutaneous SCC, we induced cutaneous SCC using a two-stage chemically induced skin carcinogenesis mouse model (Figure 3A). As shown in Figure 3B, the number of cutaneous SCC lesions is decreased in AhR-(fl/fl) Krt5-(Cre) mice, suggesting that the development of DMBA-induced cutaneous SCC depends on AhR signaling in keratinocytes. Since NHKCs increase the expression and production of Th17-related proinflammatory cytokines and chemokines *in vitro*, we evaluated these factors in established mouse cSCC. The mRNA expression of CYP1A1 (*p* = 0.002), CCL20 (*p* = 0.047), p19 (*p* = 0.029), CCL19 (*p* = 0.013), IL-36γ (*p* = 0.0076), and IL-17 (*p* = 0.0035) was significantly decreased in tumor from AhR-(fl/fl) Krt5-(Cre) mice compared with wild type mice (Figure 3C). On the other hand, there was no significant difference in mRNA expression of p40 (*p* = 0.1716), CCL22 (*p* = 0.1805), IL-36α (*p* = 0.063), and IL-36β (*p* = 0.099) in tumor from AhR-(fl/fl) Krt5-(Cre) mice compared with wild type mice.

### Expression of CCL20, IL-23, IL-36γ, and IL-17 in Cutaneous cSCC

**FIGURE 4:**
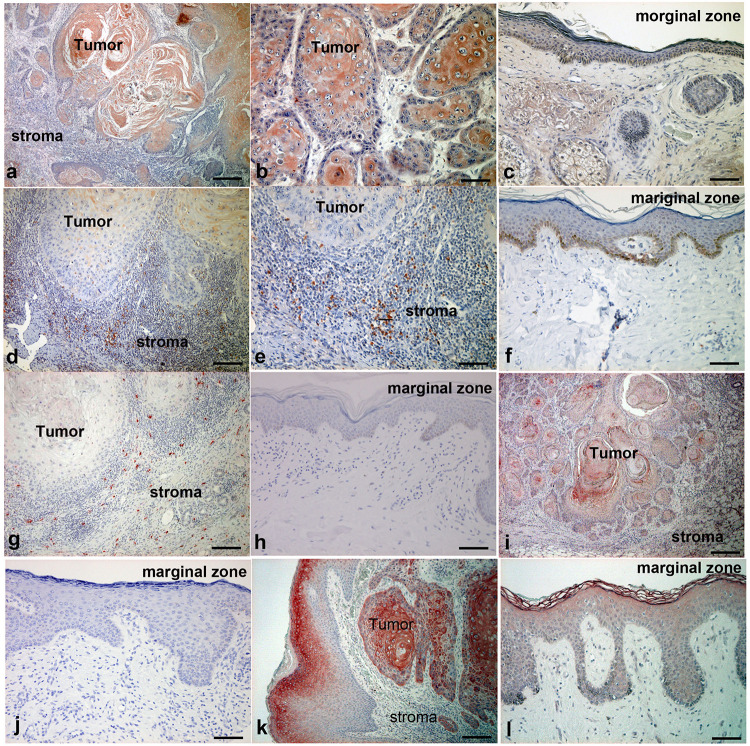
Immunohistochemical analysis of CCL20, IL-23, IL-17, IL-36γ, and IL-17R expression in lesion-affected skin of cSCC. Sections of cSCC lesions were deparaffinized and stained using anti-CCL20 **(a–c)**, anti–IL-23 **(d–f)**, anti–IL-17 **(g,h)**, anti–IL-36γ **(i,j)**, or anti–IL-17R **(k,l)** antibodies. Sections were developed with liquid permanent red. Scale bars, 100 μm. Representative specimens from 12 cases of cSCC are shown. Scale bars, 50 μm **(b,i)**, 100 μm **(c,e–l)**, and 200 μm **(a,d)**.

Considering that the cSCC mouse model described above suggested that the mRNA expression of Th17-related proinflammatory cytokines and chemokines depends on AhR signals in mouse cSCC *in vivo*, we further examined these cytokines and chemokines in cSCC patients. We employed IHC staining of CCL20, IL-23, IL-36γ, and IL-17 for samples from 10 cSCC patients. As demonstrated by the *in vitro* and *in vivo* model, atypical keratinocytes expressed CCL20 in tumor lesions of cSCC (Figures 4a,b), while normal keratinocytes at the marginal zone of the tumor did not express CCL20 (Figure 4c). Moreover, a substantial number of IL-23-producing cells were detected in the dermis of lesional skin of cSCC (Figures 4d,e), where a substantial number of IL-17-producing cells were also detected (Figure 4g). On the other hand, there were no IL-23-producing cells (Figure 4f) or IL-17-producing cells (Figure 4h) in the dermis of tumor marginal zone. These IL-23-producing cells and IL-17 producing cells were, at least in part, CD163 + tumor-associated macrophages (TAMs) and CD4 + IL-17 + Th17 cells, respectively, (Supplementary Figure 1). In addition, the expression of IL-36γ was detected in some of atypical keratinocytes (Figure 4i) but not in normal keratinocytes in the marginal zone of cSCC (Figure 4j). IL-17R is highly expressed in atypical keratinocytes in the lesional skin (Figure 4k), but only slightly expressed in the upper spiny layer of normal keratinocytes (Figure 4l).

## Discussion

Polycyclic aromatic hydrocarbons (PAHs) exert their biological effects via binding to the ligand-activated transcription factor AhR, which activates the expression of genes encoding detoxification enzymes (9). Chronic exposure of skin to PAHs, such as DMBA, induces chronic keratinocyte-specific activation of the AhR, which leads to the symptoms of atopic dermatitis with chronic inflammation (6). Another report also suggested that PAHs increase the expression of IL-5, IL-13, and IL-17 (19). These data suggest that chronic exposure of the skin to PAHs induces increased expression of both Th2 and Th17.

Recently, several reports suggested the significance of IL-17 in the development of cSCC. For example, Wu et al. reported that IL-17 signaling in keratinocytes drives IL-17-dependent sustained activation of the TRAF4-ERK5 axis, leading to keratinocyte proliferation and tumor formation in cSCC (16). In another report, IL-17 and IL-22 increased the proliferation and migration of CAL27 SCC cell lines, suggesting the contribution of IL-17 to the progression of SCC (20). Moreover, Gasparoto et al. reported the significant co-relation of IL-17 and the development of mouse cSCC (21). Furthermore, the significance of IL-17 is reported not only in cSCC, but also in other types of cancer. For example, IL-17 is positively associated with histologic grade and is described as a prognostic factor in breast cancer (22). More recently, we also reported the possible correlation between CCL20/IL-23/IL-17 axis in the development of extramammary Paget’s disease (EMPD) (18). These reports suggested the significance of IL-17 in the carcinogenesis of cancers.

IL-23 plays important roles in inducing Th17 cell proliferation (23) even in the cancer microenvironment (24), and is also known to promote growth and proliferation of human SCC of the oral cavity (25). Indeed, the significance of IL-23 is reported in various cancer species (26). For example, in UVB-induced mouse skin cancer model, several proinflammatory cytokines, including IL-23, are increased by irradiation, suggesting the relationship between IL-23 and the development of mouse AK and cSCC (27). In addition to its role in SCC, IL-23 promotes tumor progression by the inhibition of apoptosis in breast cancer cell lines (26), and levels of IL-23 and IL-23R expression are positively correlated with tumor size, tumor-node-metastasis stage, and metastasis in breast cancer (26).

Considering that the IL-23/IL-17 pathogenic axis could be an anchor cytokine signal for the development of cSCC, and that the PAHs could induce an increased expression of Th17, we hypothesized that PAHs such as DMBA and FICZ could increase the expression of these proinflammatory cytokine-related factors. Indeed, both DMBA and FICZ increased the mRNA expression of CYP1A1, CCL20, p19, and IL-36γ in NHKCs *in vitro*. In parallel to data from the *in vitro* experiments, the mRNA expression of CYP1A1, CCL20, p19, and IL-36γ, as well as IL-17 in DMBA-induced cSCC from AhR^–(fl/fl)^ Krt5^–(Cre)^ mice, is significantly decreased compared with that of wild type mice. These results suggested the significance of the IL-23/IL-17 pathogenic axis in the development of cSCC. Indeed, immunohistochemical staining for the patients with cSCC revealed that atypical keratinocytes expressed CCL20 and IL-36γ, and a substantial number of IL-23-producing cells and IL-17-producing cells were detected in the lesional skin of cSCC. Moreover, the expression of IL-17R is higher in atypical keratinocytes than in the normal keratinocytes. Taken together, our data suggested that PAHs (FICZ and DMBA) that are classically known to induce cSCC could trigger the induction of IL-17-producing cells, leading to the development of cSCC.

## Data Availability Statement

All datasets generated for this study are included in the article/Supplementary Material.

## Ethics Statement

The studies involving human participants were reviewed and approved by Tohoku University Graduate School of Medicine, Sendai, Japan (permit number: 2017-1-430) and Kagoshima Medical Center, Japan (permit number 29-2, 30-08). The patients/participants provided their written informed consent to participate in this study. The animal study was reviewed and approved by Tohoku University Graduate School of Medicine for Animal Experimentation, Sendai, Japan (permit number: 2017MdLMO-342-2).

## Author Contributions

TF and TH conception and design. TF and TH development of methodology. YS, TF, CL, and KT acquisition of data. TF analysis and interpretation of data. TF writing, review, and/or revision of the manuscript. YS, TF, KT, and SM treating patients. TF, MY, and SA study supervision. All authors contributed to the article and approved the submitted version.

## Conflict of Interest

The authors declare that the research was conducted in the absence of any commercial or financial relationships that could be construed as a potential conflict of interest.

## Supplementary Material

Supplementary Figure 1 Immunofluorescence staining of CD163 + TAMs and CD4 + T cells. Immunofluorescence staining of cSCC for IL-23 (green), CD163 (red), and DAPI (blue, nuclei; a), and IL-17 (green), CD4 (red), and DAPI (blue, nuclei). A merged image is also shown, with green and red combining into yellow. The isotype control IgG1 was stained as red or green. Representative specimens from 3 cases are shown.
