# Supplementary figures and images for: Possible Roles of Proinflammatory Signaling in Keratinocytes Through Aryl Hydrocarbon Receptor Ligands for the Development of Squamous Cell Carcinoma
Source: Front Immunol. 2020 Oct 16;11:534323. doi: 10.3389/fimmu.2020.534323 (PMC7596320; doi:10.3389/fimmu.2020.534323)

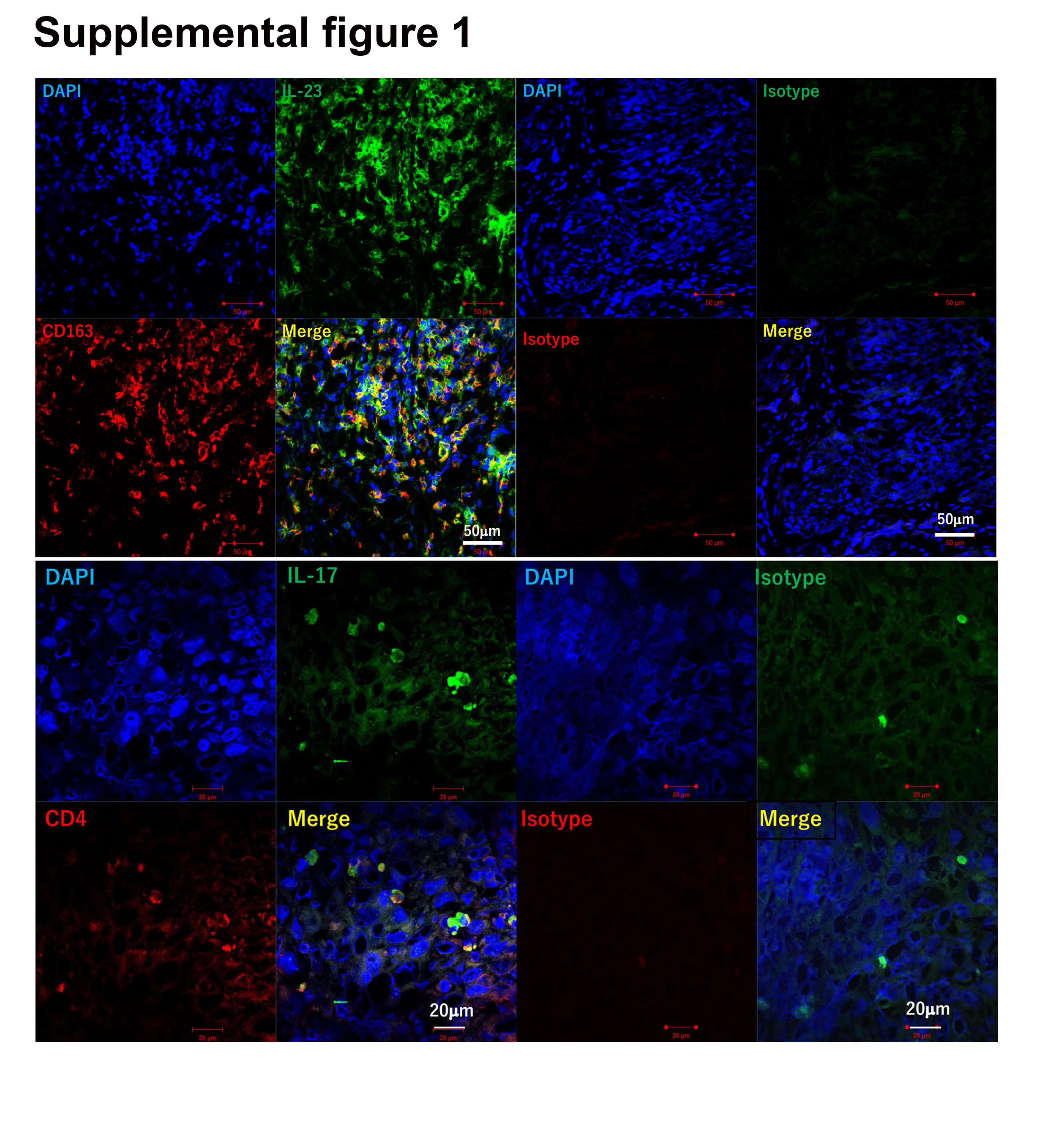

Supplement: Supplementary Figure 1 — Immunofluorescence staining of CD163 + TAMs and CD4 + T cells. Immunofluorescence staining of cSCC for IL-23 (green), CD163 (red), and DAPI (blue, nuclei; a), and IL-17 (green), CD4 (red), and DAPI (blue, nuclei). A merged image is also shown, with green and red combining into yellow. The isotype control IgG1 was stained as red or green. Representative specimens from 3 cases are shown. [file Image_1.JPEG]
